# Treatment With Liraglutide Exerts Neuroprotection After Hypoxic–Ischemic Brain Injury in Neonatal Rats *via* the PI3K/AKT/GSK3β Pathway

**DOI:** 10.3389/fncel.2019.00585

**Published:** 2020-01-30

**Authors:** Shan-shan Zeng, Jun-jie Bai, Huai Jiang, Jin-jin Zhu, Chang-chang Fu, Min-zhi He, Jiang-hu Zhu, Shang-qin Chen, Pei-jun Li, Xiao-qin Fu, Zhen-lang Lin

**Affiliations:** Department of Neonatology, The Second Affiliated Hospital and Yuying Children’s Hospital, Wenzhou Medical University, Wenzhou, China

**Keywords:** neonatal hypoxic-ischemic brain injury, liraglutide, neuroprotection, apoptosis, PI3K/AKT/GSK3β

## Abstract

Neonatal hypoxic–ischemic (HI) brain injury is a detrimental disease, which results in high mortality and long-term neurological deficits. Nevertheless, the treatment options for this disease are limited. Thus, the aim of the present study was to assess the role of liraglutide in neonatal HI brain injury in rats and investigate the associated mechanisms. The results showed that treatment with liraglutide significantly reduced infarct volume and ameliorated cerebral edema, decreased inflammatory response, promoted the recovery of tissue structure, and improved prognosis following HI brain injury. Moreover, treatment with liraglutide inhibited apoptosis and promoted neuronal survival both in the rat model and following oxygen-glucose deprivation (OGD) insult. LY294002, an inhibitor of phosphoinositide 3-kinase (PI3K), partially reversed these therapeutic effects, suggesting that the PI3K/protein kinase B (Akt) pathway was involved. In conclusion, our data revealed that treatment with liraglutide exerts neuroprotection after neonatal HI brain injury *via* the PI3K/Akt/glycogen synthase kinase-3β (GSK3β) pathway and may be a promising therapy for this disease.

## Introduction

Despite the advancements in medical technology and nursing, hypoxic–ischemic (HI) brain injury in newborns is associated with a high disability rate and mortality. Its incidence is approximately 1–8 and 26 cases per 1,000 live births in developed and developing countries, respectively (Douglas-Escobar and Weiss, 2015). Furthermore, surviving infants suffer from various degrees of neurological deficits (e.g., epilepsy, learning difficulties, cerebral palsy, and mental retardation), which require long-term or even lifelong recovery (Al-Macki et al., 2009). The physical and mental pain, as well as the high cost of HI encephalopathy, has been a constant source of stress for the families of patients with HI brain injury. Hypothermia, as a standard treatment, plays an important role in reducing the rates of mortality and disability, to some extent; however, it has a narrow therapeutic window of 6 h (Chiang et al., 2017). Therefore, the development of more effective and safer treatment strategies is warranted.

Previous studies have indicated that inflammation plays a crucial role in the deterioration and process of brain damage induced by HI injury in newborns. Once hypoxia–ischemia occurs in the neonatal brains, the development of inflammation will be promptly triggered (Hagberg et al., 2015; Solevåg et al., 2019). Immune cells, cytokines, oxidative stress, and chemokines are the main components of inflammatory processes. In addition, apoptosis, especially in neonatal brain damage after hypoxia–ischemia, is essential in various diseases of the central nervous system (Northington et al., 2011). Substantial studies confirmed that apoptosis causes delayed death of developing brain cells, leading to massive cell loss and neurodegeneration (Northington et al., 2005; Rocha-Ferreira and Hristova, 2016). Thus, lessening apoptosis serves as a useful therapeutic target following hypoxia–ischemia.

Liraglutide, a long-acting glucagon-like peptide-1 (GLP-1) analogue, has been widely used in the clinical treatment of type 2 diabetes owing to its efficacy and safety (Scott, 2014). GLP-1 is a visceral-derived insulin-stimulating hormone, which is secreted into the bloodstream and bound to the GLP-1 receptor (GLP-1R; Vilsboll, 2009). Additionally, GLP-1R plays a role in blood glucose homeostasis and is extensively expressed in the central nervous system, including neurons, cell bodies, and dendrites in the brain (Hamilton and Hölscher, 2009; Cork et al., 2015). Compared with other GLP-1 analogues, liraglutide has a 13-h half-life, binding to serum albumin to avoid the proteolytic degradation of dipeptide peptide-4. Moreover, liraglutide has been shown to cross the blood–brain barrier (Hunter and Hölscher, 2012). Numerous studies have suggested that liraglutide exerts neuroprotective effects against injury and diseases of the nervous system, such as Alzheimer’s disease (McClean et al., 2011; McClean and Hölscher, 2014), Parkinson’s disease (Aviles-Olmos et al., 2014; Badawi et al., 2017), acute ischemic stroke (Yang et al., 2016; Dong et al., 2017), and traumatic brain injury (DellaValle et al., 2014; Hakon et al., 2015). A recently published study indicated that liraglutide could alleviate chronic inflammation and mitochondrial stress induced by status epilepticus (Wang et al., 2018). Thus, far, the specific mechanism through which liraglutide exerts its effects on neonatal HI brain injury remains uncertain.

The phosphoinositide 3-kinase (PI3K)/protein kinase B (Akt) signaling pathway participates in a diversity of physiological and pathogenetic processes, such as cell apoptosis, proliferation, and differentiation. Some research studies have demonstrated that this signaling pathway protects neurons from apoptosis in different brain diseases (Ye et al., 2019). Glycogen synthase kinase-3β (GSK3β), one of the downstream targets of activated Akt, has been closely associated with energy metabolism, body shape development, and nerve cell development. Activation of GSK3β further exacerbates cell damage by increasing the activity of caspase 3 cleavage, which is an early step in apoptosis (King et al., 2001). Moreover, a recent study showed that the PI3K/Akt/GSK3β signaling pathway plays a vital role in neuroprotection after intracerebral hemorrhage (Chen et al., 2019b).

In this study, we examined the neuroprotective role of liraglutide in neonatal brain damage induced by HI injury, and we determined whether the PI3K/Akt/GSK3β signaling pathway is involved in this process.

## Materials and Methods

### Animals and Ethical Permission

All animal experimental procedures and care were performed according to the National Institutes of Health Guide for the Care and Use of Laboratory Animals and approved by the Animal Care and Use Committee of Wenzhou Medical University (ethic number: wydw2014-0058). Sprague–Dawley rats (weight: 200–250 g) were provided by the Animal Center of the Chinese Academy of Science (Shanghai, China). Adult rats were freely crossbred to deliver offspring for the follow-up studies. They were housed in an individual environment under specific pathogen-free conditions and a 12-h light/dark cycle at 23 ± 2°C and 60 ± 10% humidity, with *ad libitum* access to standard food and fresh water.

### Neonatal Hypoxic–Ischemic Brain Injury Model and Administration of Drug

The neonatal HI brain injury model was generated on postnatal day 7 (P 7) using male rat pups, as described by Vannucci and Vannucci (2005). In brief, the 7-days-old pups were fully anesthetized with 3% isoflurane and sustained with 1% isoflurane. Subsequently, they underwent left common carotid artery ligation in 5 min and recovered for 2 h in their dam after surgery. Following sufficient rest, the pups were placed in a chamber for 2.5 h, in an environment of a humidified gas mixture (8% oxygen and 92% nitrogen) at a flow rate of 3 L/min. A water bath of 37.5°C was placed underneath the chamber to maintain a constant temperature. Pups in the sham group were not subjected to ligation of the common carotid arteries or hypoxic conditions.

Clinical-grade liraglutide was purchased from Novo Nordisk (Princeton, NJ, USA) and dissolved in sterile 0.9% normal saline. The liraglutide-treated HI group received different doses of liraglutide (i.e., 100, 200, or 400 μg/kg) immediately after hypoxia through intraperitoneal injection at 24-h intervals until the animals were euthanized, to determine the most effective dose. Meanwhile, the pups in the vehicle-treated HI group received an equal volume of sterile 0.9% normal saline. The PI3K inhibitor LY294002 (Selleck, Shanghai, China) was dissolved in 1% dimethyl sulfoxide to further evaluate whether liraglutide activated the PI3K/Akt/GSK3β pathway. Five microliters of LY294002 (50 nmol/kg; Ye et al., 2019) was administered *via* intracerebroventricular injection 30 min prior to HI using a stereotaxic apparatus (RWD, Shenzhen, China). LY294002 was injected into the lateral ventricle (2 mm rostral, 1.5 mm outside the bregma, and 2.5 mm below the skull; Zhou et al., 2017) at a speed of 1 μl/min. After injection, the needle remained in place for another 10 min and was subsequently extracted at a rate of 1 mm/min.

### Infarct Volume Measurement

Staining with 2,3,5-triphenyltetrazolium chloride (TTC) was used as previously described (Tian et al., 2013) to measure the infarct volume relieved by the administration of drug. After HI injury (24 h), pups from each group were anesthetized and perfused with 0.9% cold normal saline. The brains were immediately stored at −80°C for 6 min and sectioned into coronal slices (thickness: 2 mm). Subsequently, the brain slices were immersed in a 1% TTC (Sigma–Aldrich, St. Louis, MO USA) solution in the dark for 20 min at 37°C and fixed in 4% paraformaldehyde (PFA) overnight. Brain infarct volumes were calculated using the ImageJ software (National Institutes of Health, Bethesda, MD, USA).

### Water Content in the Brain

The 7-days-old rats under deep anesthesia were sacrificed 24 h after HI injury for brain analyses. The brains from each group were rapidly removed, and the hemispheres were separated into the ipsilateral and contralateral. The injured hemispheres were weighted to measure wet weight (accuracy to 0.1 mg). Subsequently, each hemisphere was placed in an oven (100°C) for 72 h to calculate the dry weight (accuracy to 0.1 mg) as previously described (Zhang et al., 2016). The degree of brain edema was calculated according to the wet/dry method: percent brain water = [(wet weight − dry weight)/wet weight] × 100%.

### Quantitative Real-Time Reverse Transcription–Polymerase Chain Reaction

Total RNA was extracted from the samples using the TriPure Isolation Reagent (Roche, South San Francisco, CA, USA) according to the instructions provided by the manufacturer. NanoDrop spectrometry (Thermo Fisher Scientific, Waltham, MA, USA) was used to quantify the concentration of total RNA; only samples with an optical density 260/280 ratio >1.8 were selected. RNA (0.5 μg) was used to synthesize the cDNA utilizing the PrimeScript™ RT Reagent Kit (TaKaRa, Kusatsu, Japan) and Bio-Rad MyCycler™ thermal cycler for the reverse transcription–polymerase chain reaction. With the use of the SYBR Green PCR Master Mix (Bio-Rad, Hercules, CA, USA), samples were amplified by the real-time polymerase chain reaction system. β-Actin was used for standardization. The forward and reverse primer sequences are shown in Table 1. Subsequently, we calculated the fluorescence threshold value (cycle threshold value) *via* the SDS Enterprise Database software. The cycle threshold values of the correlative genes were normalized to those of β-actin in the same sample. Next, the levels of gene expression (fold change) in the vehicle- and liraglutide-treated groups were measured and compared with those reported in the sham group.

**Table 1:** Primers used in the studies.

Gene	Forward primers	Reverse primers
TNF-α	TACTCCCAGGTTCTCTTCAAGG	GGAGGCTGACTTTCTCCTGGTA
IL-18	AAACCCGCCTGTGTTCGA	TCAGTCTGGTCTGGGATTCGT
IL-1β	CACCTCTCAAGCAGAGCACAG	GGGTTCCATGGTGAAGTCAAC
IL-6	GAGTTGTGCAATGGCAATTC	ACTCCAGAAGACCAGAGCAG
Inos	AGGCCACCTCGGATATCTCT	GCTTGTCTCTGGGTCCTCTG
β-Actin	AAGTCCCTCACCCTCCCAAAAG	AAGCAATGCTGTCACCTTCCC

*Note: TNF-α, tumor necrosis factor-alpha; IL, interleukin; iNOS, inducible nitric oxide synthase*.

### Histological Staining

After HI injury (24 h or 7 days), the rats were anesthetized and subjected to cardiac perfusion with 20 ml of normal saline. Subsequently, they were perfused with 4% PFA for better fixation. The brains of the rats were fixed in 4% PFA for 12 h at 4°C. Next, brains were embedded in paraffin and sectioned into 5-μm slices along the coronal plane for subsequent staining after dehydration with graded ethanol and xylene. Hematoxylin and eosin (H&E) staining and Nissl staining were used based on the protocol provided by the manufacturer to assess histopathological changes. Meanwhile, different organs (e.g., the heart, liver, lungs, pancreas, and kidneys) were stained with H&E for the histopathological assessment of side effects following the previous steps. The bright-field images were captured with a light microscope (Olympus IX73, Tokyo, Japan). Nissl-stained brain sections were analyzed through the ImageJ software to quantify the damage caused to neurons.

### Immunofluorescence and Immunohistochemistry

Coronal 5-μm sections were selected for immunofluorescence (7 days after HI injury) and immunohistochemical (24 h after HI injury) analyses. After deparaffinization and rehydration, sections were boiled in citrate buffer for 20 min for antigen retrieval. The slices were incubated in 3% hydrogen peroxide for 15 min to block the endogenous peroxidase activity. Subsequently, they were blocked with 5% bovine serum albumin in 10 mM of phosphate-buffered saline (PBS) for 30 min at 37°C. For the immunofluorescence analysis, the sections were incubated with primary antibodies targeting the following protein: tumor necrosis factor-alpha (TNF-α; 1:200, AF7014; Affinity Biosciences, Cincinnati, OH, USA). The sections were washed thrice with PBS and treated with the Alexa Fluor 488 goat anti-rabbit secondary antibody (1:1,000, Abcam, Cambridge, UK) for 1 h at 37°C in a dark humidified chamber. The primary antibodies were diluted in 10 mM of PBS containing 1% bovine serum albumin and 0.2% Triton X-100. After being washed thrice with PBS, the sections were treated with the goat anti-rabbit IgG (H + L) DyLight 405 sary antibody (1:200, BS10012; Bioworld) for 1 h at 37°C in a dark humidified chamber. The sections were washed thrice with PBS Tween 20, and the nuclei were labeled with 4′,6-diamidino-2-phenylindole for 7 min. All images were captured using a fluorescence microscope (Olympus, Tokyo, Japan). For the immunohistochemical analysis, the sections were incubated with antibodies against microtubule-associated protein 2 (MAP-2; 1:200, sc-20172; Santa Cruz Biotechnology, Dallas, TX, USA), myelin basic protein (MBP; 1:200, sc-13914; Santa Cruz Biotechnology, Dallas, TX, USA), and phospho-GSK3 beta (Ser9; p-GSK3β; 1:200, AF2016; Affinity Biosciences, Cincinnati, OH, USA) at 4°C overnight. After being washed thrice with PBS, the sections were treated with donkey anti-goat secondary antibody (1:1,000, sc-2020; Santa Cruz Biotechnology, Dallas, TX, USA) for 2 h at 37°C. Subsequently, the reaction was colored with 3,3′-diaminobenzidine. The images were captured using a light microscope (Olympus, Tokyo, Japan).

### Cell Culture and Drug Treatment

PC12 cells were purchased from the Type Culture Collection of the Chinese Academy of Sciences (Shanghai, China). They were cultured in Dulbecco’s modified Eagle’s medium (DMEM; Gibco, Grand Island, NY, USA) supplemented with 10% fetal bovine serum (Gibco, Grand Island, NY, USA), 100 U/ml of streptomycin, and 100 U/ml of penicillin in an environment of 5% carbon dioxide at 37°C. After attachment, the cells were treated with 50 ng/ml of nerve growth factor (NGF; Promega, Madison, WI, USA) and cultured in serum-free DMEM for 6 days to induce neurite formation. Subsequently, cells were subjected to an *in vitro* oxygen-glucose deprivation (OGD) insult for 8 h with a mixed gas consisting of 95% nitrogen and 5% carbon dioxide. Meanwhile, the culture medium was replaced with glucose-free DMEM. LY294002 (10 μM) was used for 30 min before other treatment. After the OGD, the culture medium was replaced with original medium, and liraglutide was added. The plates were continually cultured in a normoxic incubator for reoxygenation in an atmosphere of 5% carbon dioxide at 37°C for 24 h. The cells of the control group were cultured under normal conditions without oxygen or glucose deprivation.

### Cell Counting Kit-8 Assay

Cell Counting Kit-8 (CCK8; C0038; Beyotime, Shanghai, China) assays were used to evaluate cell viability, assess whether liraglutide could alleviate OGD-induced cell death, and determine the optimal concentration. PC12 cells were plated in 96-well plates at a density of 2 × 10^4^ cells/well. Different doses of liraglutide (0, 10, 100, and 1,000 nM) were added after OGD. Next, 10 μl of CCK-8 solution per well was added after reoxygenation, and the optical density was measured using a microplate reader (Tecan Group Limited, Männedorf, Switzerland) at 490 nm.

### Terminal Deoxynucleotidyl Transferase dUTP Nick End Labeling Staining

The *in situ* Cell Death Detection Kit (Roche, South San Francisco, CA, USA) was used to detect apoptotic DNA fragmentation. *In vivo*, terminal deoxynucleotidyl transferase dUTP nick end labeling (TUNEL) staining was performed 24 h after HI injury. The brain sections (thickness: 5 μm) obtained 24 h after HI injury were deparaffinized and rehydrated. Subsequently, they were incubated with 20 μg/ml of proteinase K working solution (ST533; Beyotime, Shanghai, China) for 20 min at 37°C. After OGD injury for 24 h, PC12 cells were cultured in six-well plates with 4% PFA *in vitro* for 1 h, and 0.1% Triton X-100 was incubated in 0.1% sodium citrate for 2 min on ice. After being washed thrice with PBS, the sections and cells were incubated with a TUNEL reaction mixture in a dark humidified chamber for 1 h at 37°C. The negative group was treated by omitting the terminal deoxynucleotidyl transferase enzyme. The sections were washed thrice with PBS, followed by incubation with 4′,6-diamidino-2-phenylindole for 7 min at room temperature. All apoptotic changes were detected using a fluorescence microscope (Olympus, Tokyo, Japan). The apoptotic cells were characterized by green fluorescence, and 4′,6-diamidino-2-phenylindole labeled the nuclei with blue color, according to the instructions provided by the manufacturer. The ratio of the number of apoptotic cells to the total number of cells represented the apoptosis index, which was analyzed using the ImageJ software.

### Western Blotting

Rats were decapitated under deep anesthesia 24 h and 7 days after HI injury, and brain tissue was immediately stored at −80°C until analysis. The brain tissue was homogenized with radioimmunoprecipitation assay buffer (P0013B; Beyotime) and further centrifuged at 12,000 rpm for 30 min at 4°C. The extraction was initially quantified with bicinchoninic acid reagents (P0012S; Beyotime). Subsequently, the protein concentrations were quantified using bicinchoninic acid reagents. Equivalent amounts of protein (50 μg) were loaded and separated through 6% or 10% sodium dodecyl sulfate–polyacrylamide gel electrophoresis and transferred onto polyvinylidene difluoride membranes (Bio-Rad, Hercules, CA, USA). After being blocked for 2 h at room temperature, the membranes were incubated with the primary antibodies overnight at 4°C. The primary antibodies used included B-cell lymphoma-2 (Bcl-2; 1:1,000, #2876; Cell Signaling Technology), cleaved caspase 3 (1:1,000, 9664S; Cell Signalling Technology, Danvers, MA, USA), TNF-α (1:1,000, AF7014; Affinity Biosciences, Cincinnati, OH, USA), MAP-2 (1:1,000, #4542S; Cell Signalling Technology, Danvers, MA, USA), MBP (1:1,000, ab40390; Abcam, Cambridge, UK), Akt (1:1,000, #9272; Cell Signalling Technology, Danvers, MA, USA), phospho-Akt (Ser473; 1:1,000, #9271; Cell Signalling Technology, Danvers, MA, USA), GSK3β (1:1,000, #9315; Cell Signalling Technology, Danvers, MA, USA), phospho-GSK3β (ser9; 1:1,000, #5558; Cell Signalling Technology, Danvers, MA, USA), and β-actin (1:5,000, 66009–1-Ig; Proteintech). After being washed thrice in tris-buffered saline and Tween 20, the membranes were incubated with the appropriate secondary antibodies, namely, goat anti-rabbit IgG (1:2,000, #7074, Cell Signalling Technology, Danvers, MA, USA) or goat anti-mouse IgG (1:2,000, #7076; Cell Signalling Technology, Danvers, MA, USA) for 1 h at room temperature. The signals were detected using a ChemiDoc XRS + Imaging System (Bio-Rad, Hercules, CA, USA). All experiments were repeated at least in triplicate, and the gray values of the bands were analyzed using the Image Lab 5.0 software (Bio-Rad, Hercules, CA, USA).

### Longa Assessment

Three weeks post HI injury, neurobehavioral tests were performed to evaluate the injury or improvement of nerve function after the administration of drug in rats. There are five levels of neurological examination: 0, normal function, no neurological deficit; 1, the right forepaw could not be fully extended, mild neurological deficit; 2, keep turning to the right while walking, moderate neurological deficit; 3, body leaning to the right while walking, severe neurological deficit; and 4, unable to walk spontaneously, unconsciousness. All neurobehavioral tests, using a double-blind procedure, were performed in triplicate.

### Berderson Behavioral Test

At 28 days of age, the Berderson behavioral test was performed to evaluate the degree of contralateral paralysis in rats. Briefly, the rats were lifted 1 m in the air to observe the flexion of the forelimbs, and the score was as follows: 0, rats extended the forelimbs to the ground without neurological deficits; 1, rats sustained flexion injury to the hemisphere of the affected limb in different postures including mild wrist flexion, shoulder adduction elbow extension to severe postures, complete wrist and elbow flexion, shoulder rotation, and adduction; and 2, rats were placed on a large paperboard where they could grip tightly. Gentle pressure was used behind the shoulder until the forelimbs slid several inches in each direction. Rats with normal or mild functional impairment exhibited similar lateral resistance to slippage; and 3, rats consistently circled toward the paretic side. All neurobehavioral tests, using a double-blind procedure, were performed in triplicate.

### Statistical Analysis

The quantitative results were presented as the mean ± standard error of the mean from three independent experiments. Student’s *t*-test was used to assess the statistical significance between two experimental groups. For the comparison of more than two groups, one-way analysis of variance was used followed by Tukey’s test. All statistical analyses were performed using GraphPad Prism 6.0 (GraphPad Software Inc., San Diego, CA, USA). *P* < 0.05 denoted statistical significance.

## Results

### Treatment With Liraglutide Reduced Infarct Volume and Ameliorated Cerebral Edema

**Figure 1:**
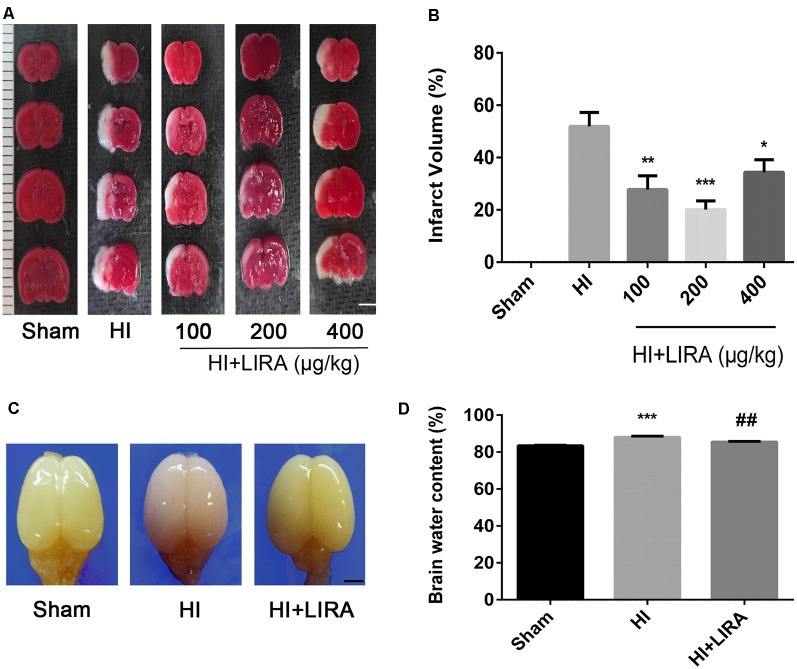
Treatment with liraglutide attenuated the infarct volume and cerebral edema after hypoxic–ischemic (HI) brain injury. **(A)** Representative 2,3,5-triphenyltetrazolium chloride (TTC)-stained coronal brain sections 24 h after HI brain injury. Scale bar = 1 mm. **(B)** Quantitative analysis of infarct volume. *P < 0.05, **P < 0.01, and ***P < 0.001 vs. the HI group. Values represent the mean ± SEM. *n* = 4. **(C)** The general shape of the brain 24 h after HI. *n* = 5. Scale bar = 1 mm. **(D)** Quantification of the water content in the ipsilateral brain hemisphere 24 h after HI. ***P < 0.001 vs. the sham group. ^##^*P* < 0.01 vs. the HI group. Values are presented as mean ± SEM. *n* = 5.

Liraglutide (100, 200, and 400 nmol/kg) was administered through intraperitoneal injection immediately after HI brain injury to examine its role in this process and determine the most effective dose. According to the quantitative analysis of the TTC-stained sections, all three doses effectively reduced infarct volume, and the 200 nmol/kg resulted in the most pronounced effect. Therefore, 200 nmol/kg of liraglutide was used in all following experiments as the standard treatment (Figures 1A,B).

Regarding cerebral edema, the shape of brains and water content in the brains were examined 24 h after HI. Evidently, owing to edema, the ipsilateral brains obtained from the HI group were larger compared with those obtained from the sham group and HI + Liraglutide group (Figure 1C). The results of the water content analysis indicated that the content in the ipsilateral brain of the HI group was significantly increased in contrast to that measured in the sham group. Of note, the HI + Liraglutide group, compared with the HI group, exhibited a marked reduction (Figures 1C,D).

### Treatment With Liraglutide Decreased the Expression of Inflammatory Factors at Both the mRNA and Protein Levels

**Figure 2:**
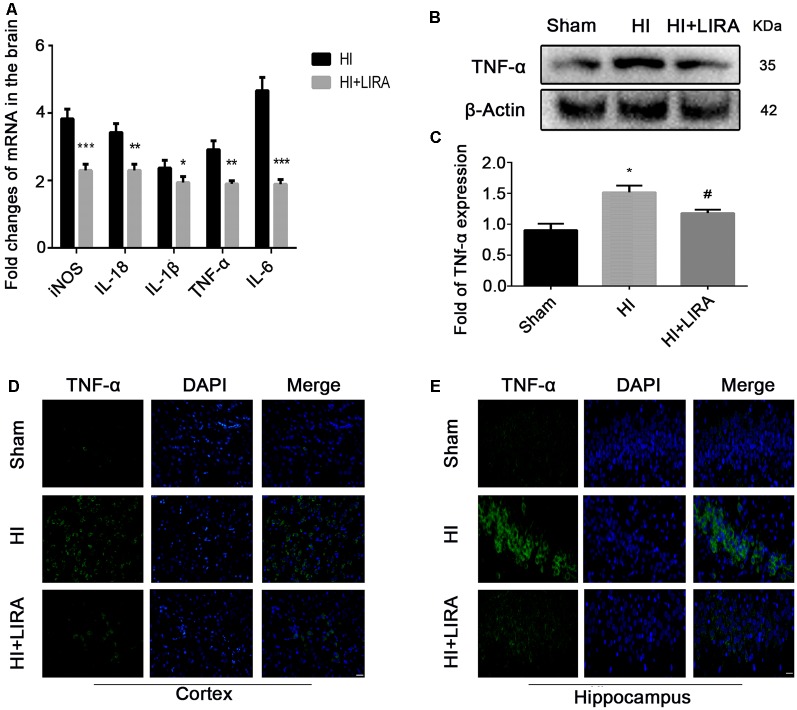
Treatment with liraglutide decreased the inflammatory response at both the mRNA and protein levels. **(A)** The levels of mRNA expression in brain tissue 24 h after hypoxic–ischemic (HI) injury normalized to those of β-actin for each sample. *P < 0.05, **P < 0.01, and ***P < 0.001 vs. the HI group. *n* = 5. Values are presented as mean ± SEM. *n* = 5. Protein expression level **(B)** and quantification data **(C)** of tumor necrosis factor-alpha (TNF-α) 24 h after HI injury. *P < 0.05 vs. the sham group. ^#^*P* < 0.05 vs. the HI group. Values are presented as mean ± SEM. *n* = 3. Representative images of immunofluorescence staining of TNF-α (green) in the cortex **(D)**, hippocampus **(E)**, and nucleus (blue) labeled with DAPI. Scale bar = 25 μm.

Several inflammatory markers were assessed 24 h after HI injury using real-time reverse transcription–polymerase chain reaction to verify whether treatment with liraglutide can reduce the release of inflammatory cytokines. The mRNA expression levels of TNF-α, IL-18, IL-1β, IL-6, and cyclooxygenase-2 in the HI group were markedly increased compared with those detected in the sham group. Nevertheless, the mRNA levels of these inflammatory factors were significantly decreased in the HI + Liraglutide group (Figure 2A). Additionally, the levels of TNF-α in the sham group were the lowest and increased after the HI insult (Figure 2B). Furthermore, the protein expression of TNF-α in the HI + Liraglutide group was markedly inhibited (Figures 2B,C). In the sham group, immunofluorescence staining of TNF-α showed low expression in both the cortex (Figure 2D) and hippocampus (Figure 2E). Positive immunoreactivity for TNF-α was strongly found in the HI group, indicating a prominent release of inflammatory cytokines in the cortex and hippocampus. In contrast to the HI group, we observed a lower fluorescence signal for TNF-α.

### Long-Term Neuroprotective Effects of Treatment With Liraglutide

**Figure 3:**
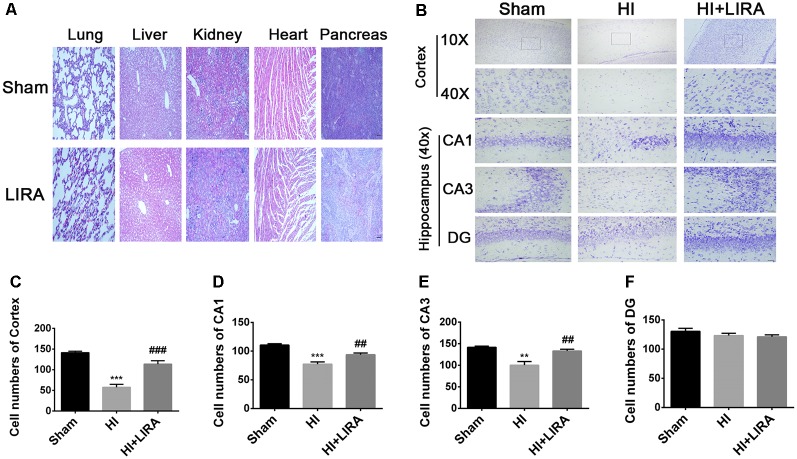
Treatment with liraglutide reduced the damage in tissue structure and neuron loss after HI injury. **(A)** Representative images of H&E staining of the heart, liver, lung, pancreas, and kidney at day 7 post-treatment with liraglutide. Scale bar = 50 μm. **(B)** Representative images and quantification data **(C–F)** of Nissl staining in the cortex and hippocampus of CA1, CA3, and dentate gyrus at day 7 post HI injury. Scale bar = 25 μm, 100 μm. Values are presented as mean ± SEM. *n* = 5. **P < 0.01 and ***P < 0.001 vs. the sham group. ^##^*P* < 0.01 and ^###^*P* < 0.001 vs. the HI group. Values are presented as mean ± SEM. *n* = 5.

Meanwhile, H&E staining was conducted to investigate whether liraglutide exerts any effect on the important organs (in terms of pathology) after 7 days of continuous administration (Figure 3A). There was no apparent difference between the sham group and liraglutide-treated group. To further investigate the neuroprotection of treatment with liraglutide against HI-induced brain injury, rats from each group were sacrificed to observe the brain atrophy and tissue loss at day 14.

Seven days post HI insult, Nissl staining was used to evaluate the histopathological and neuronal transformation in the ipsilateral cortex and hippocampus. We counted the numbers of Nissl bodies in the injured hemisphere to investigate the protective effect of liraglutide. The Nissl bodies were large and abundant and remained well arranged around the nuclei. In contrast, after HI injury, we observed decreased and disorganized neurons and even the absence of neurons (Figures 3B–F).

**Figure 4:**
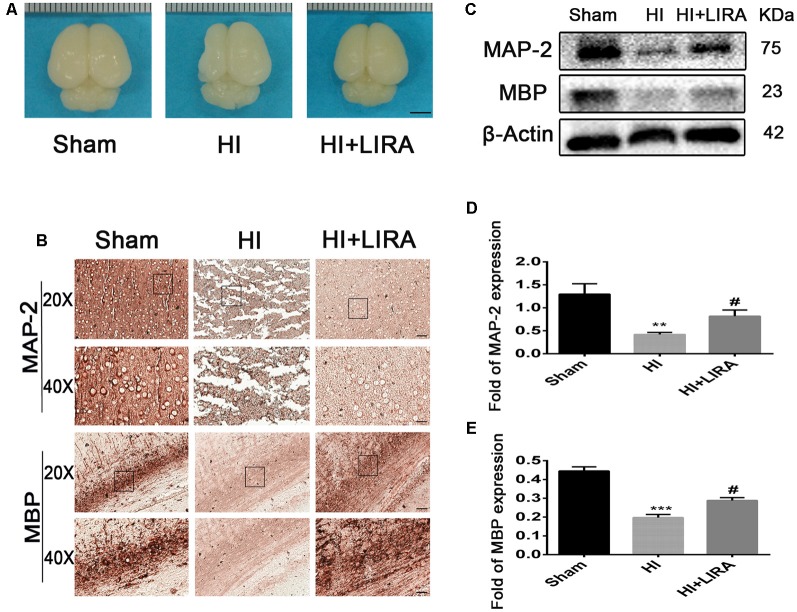
Treatment with liraglutide decreased brain atrophy, enhanced axonal repair, and accelerated remyelination after HI injury. **(A)** General observation of rat brains from each group at day 7 post HI injury. Scale bar = 1 mm. **(B)** Representative images of immunohistochemical staining for microtubule-associated protein 2 (MAP-2) and myelin basic protein (MBP). Scale bar = 25 μm, 50 μm. Protein expression level **(C)** and quantification data of MAP-2 **(D)** and MBP **(E)** at day 7 post HI injury. **P < 0.01 and ***P < 0.001 vs. the sham group. ^#^*P* < 0.05 vs. the HI group. Values are presented as mean ± SEM. *n* = 3.

Moreover, after treatment with liraglutide, the brain atrophy was ameliorated (Figure 4A). And there was an increase in the number of neurons and Nissl bodies. Immunohistochemical analysis of MAP-2 (a biomarker for neurons) and MBP (a biomarker for oligodendrocytes) and western blotting were applied to determine whether liraglutide was able to accelerate remyelination and axonal repair in neonatal pups. As shown in Figures 4B–E, we found the lowest protein expression of MAP-2 and MBP in the vehicle-treated HI group. In contrast, there was a significant increase in the level of both proteins after treatment with liraglutide. Correspondingly, the results of the immunohistochemical analysis showed the same trend. Both MAP-2 and MBP were weakly expressed in the HI group, whereas treatment with liraglutide upregulated the expression of the positive area.

### Treatment With Liraglutide Exerted Neuroprotection by Activating the PI3K/Akt/GSK3β Pathway

The PI3K inhibitor LY294002 was used to evaluate whether liraglutide activated the PI3K/Akt/GSK3β pathway. According to the quantitative analysis, the HI + Liraglutide + LY294002 group, compared with the HI + Liraglutide group, showed a markedly increased volume of cerebral infarction (Figures 6A,B) and water content in the brain (Figures 6C,D). In addition, western blotting was performed to measure the levels of proteins involved in the PI3K/Akt/GSK3β pathway. The levels of p-Akt and p-GSK3β in the HI group were lower than those reported in the sham group, and they increased after the HI insult. However, the levels of both proteins were increased compared with those recorded in the HI group, whereas protein expression in the HI + Liraglutide + LY294002 group was dramatically inhibited (Figure 6E). The levels of Akt and GSK3β among the four groups were not statistically significant (Figure 6F). Moreover, the number of dying cells (i.e., shrunken nucleus and decreased neuronal density) was significantly increased in the HI + Liraglutide + LY294002 group (Figure 6G). Correspondingly, the results of the immunohistochemical analysis showed the same trend. P-GSK3β was weakly expressed in the HI group, whereas treatment with liraglutide upregulated the number of positive cells of p-GSK3β. Moreover, the number of positive cells of p-GSK3β was obviously decreased in the HI + Liraglutide + LY294002 group (Figure 6H). To eliminate effects caused by LY294002 only, we did experiments to compare HI + LY294002 group with the HI group. There were no statistically significant differences between the two groups in infarct volume, degree of brain edema, tissue structure, prognosis *in vivo* and cell viability *in vitro* (Supplementary Figure S1). These data revealed that the neuroprotection of liraglutide involved the activation of the PI3K/Akt/GSK3β pathway.

**Figure 5:**
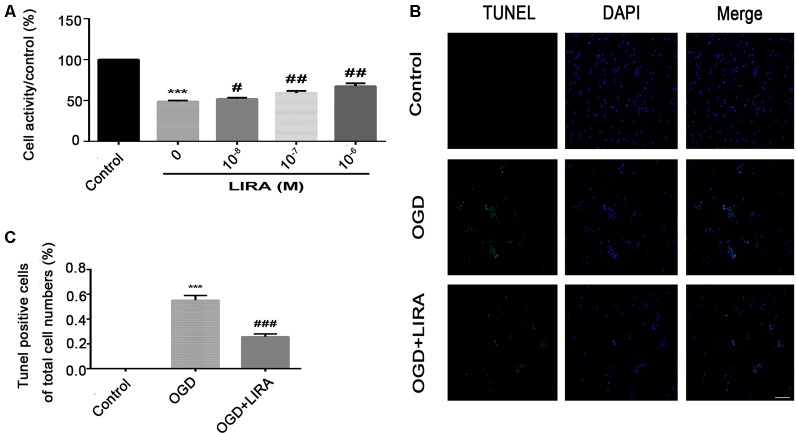
Treatment with liraglutide alleviated cell death and apoptosis induced by oxygen-glucose deprivation (OGD). **(A)** Cell viability after reoxygenation for 24 h following OGD for 8 h with different doses of liraglutide. ***P < 0.001 versus the control group. ^#^*P* < 0.05 and ^##^*P* < 0.01 versus the OGD group. Values are presented as mean ± SEM. *n* = 3. **(B)** Representative images of terminal deoxynucleotidyl transferase dUTP nick end labeling (TUNEL) staining (green); the nucleus (blue) was labeled with DAPI 24 h HI injury. Scale bar = 50 μm. **(C)** Quantification data of TUNEL staining. ***P < 0.001 vs. the control group. ^###^*P* < 0.001 vs. the OGD group. Values presented as mean ± SEM. *n* = 4.

**Figure 6:**
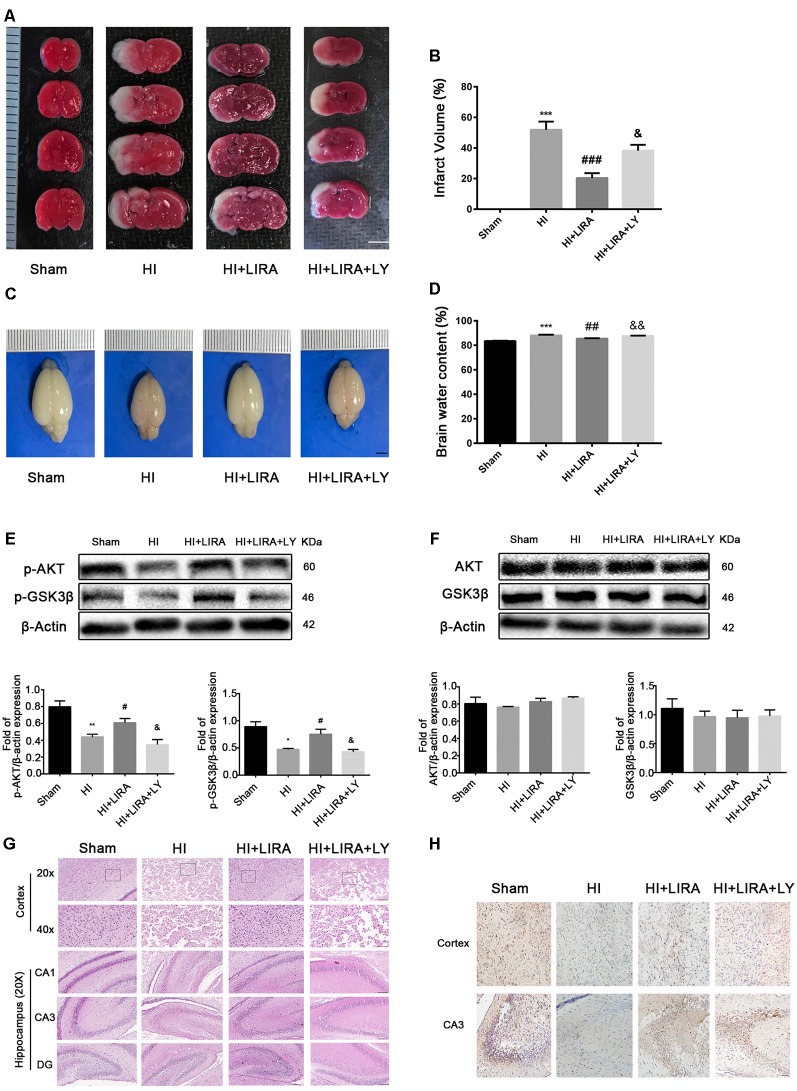
Treatment with liraglutide (LIRA) exerted neuroprotection by activating the phosphoinositide 3-kinase (PI3K)/protein kinase B (Akt)/glycogen synthase kinase-3β (GSK3β) pathway. **(A)** Representative TTC-stained coronal brain sections 24 h after HI. *n* = 4. Scale bar = 1 mm. **(B)** Quantitative analysis of infarct volume. ***P < 0.001 vs. the sham group. ^###^*P* < 0.001 vs. the HI group. ^&^*P* < 0.05 vs. the HI + LIRA group. Values are presented as mean ± SEM. *n* = 4. **(C)** The general shape of the brain 24 h after HI. *n* = 5. Scale bar = 1 mm. **(D)** Quantification of water content in the ipsilateral brain hemisphere 24 h after HI. ***P < 0.001 vs. the sham group. ^##^*P* < 0.01 vs. the HI group. ^&&^*P* < 0.01 vs. the HI + LIRA group. Values are presented as mean ± SEM. *n* = 5. **(E)** Protein expression level and quantification data of p-AKT and p-GSK3β 24 h after HI injury. *P < 0.05 and **P < 0.01 vs. the sham group. ^#^*P* < 0.05 vs. the HI group. ^&^*P* < 0.05 vs. the HI + LIRA group. Values are presented as mean ± SEM. *n* = 3. **(F)** Protein expression level and quantification data of AKT and GSK3β 24 h after HI injury. Values are presented as mean ± SEM. *n* = 3. **(G)** Representative images of H&E staining in the cortex and hippocampus of CA1, CA3, and dentate gyrus at day 7 post HI injury. Scale bar = 25 μm, 50 μm. **(H)** Representative images of immunohistochemical staining for p-GSK3β. Scale bar = 50 μm.

### The Anti-Apoptotic Effect of Liraglutide Could be Inhibited by LY294002 Both *in vivo* and *in vitro*

**Figure 7:**
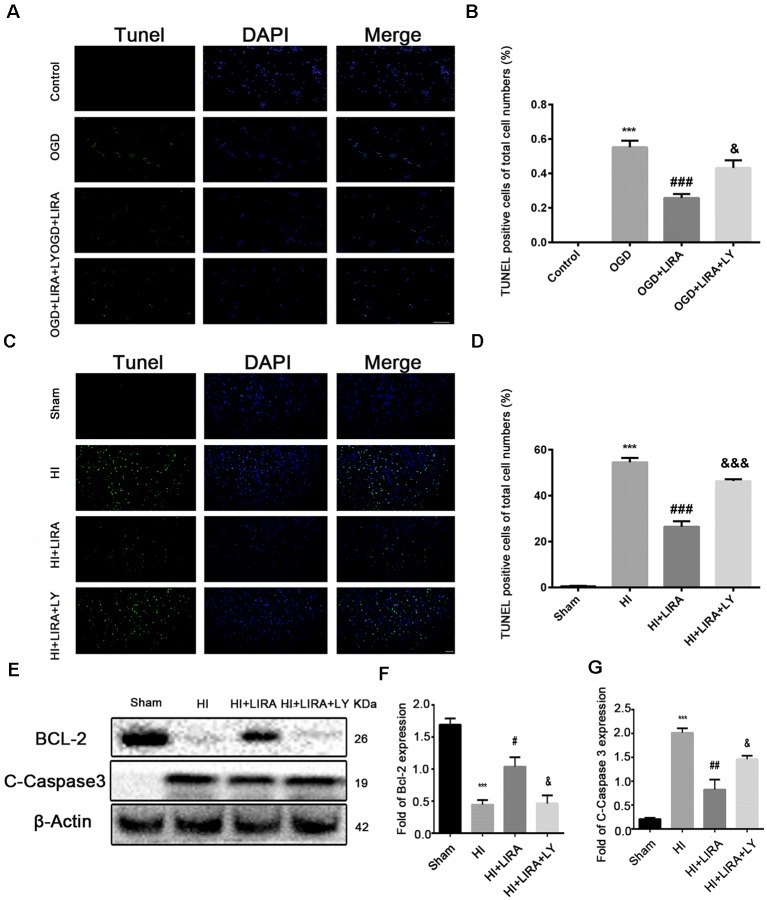
The anti-apoptotic effect of liraglutide (LIRA) could be inhibited by LY294002 both *in vivo* and *in vitro*. **(A)** Representative images of terminal deoxynucleotidyl transferase dUTP nick end labeling (TUNEL) staining (green) and nucleus (blue) were labeled with DAPI after OGD injury in PC12 cells. Scale bar = 50 μm. **(B)** Quantification data of TUNEL staining *in vitro*. ***P < 0.001 vs. control group.^ ###^*P* < 0.001 vs. the OGD group. ^&&^*P* < 0.01 vs. the OGD + LIRA group. Values are presented as mean ± SEM. *n* = 4. **(C)** Representative images of TUNEL staining (green); the nucleus (blue) was labeled with DAPI 24 h post hypoxic–ischemic (HI) injury in the animal model. Scale bar = 50 μm. **(D)** Quantification data of TUNEL staining *in vivo*. ***P < 0.001 vs. the sham group. ^###^*P* < 0.001 vs. the HI group. ^&&&^*P* < 0.001 vs. the HI + LIRA group. Values are presented as mean ± SEM. *n* = 5. Protein expression level **(E)** and quantification data of Bcl-2 **(F)** and C-Caspase 3 **(G)** 24 h after HI injury. ***P < 0.001 vs. the sham group. ^#^*P* < 0.05 and ^##^*P* < 0.01 vs. the HI group. ^&^*P* < 0.05 vs. the HI + LIRA group. Values are presented as mean ± SEM. *n* = 3.

The CCK8 assay was performed to confirm whether treatment with liraglutide protects against injury induced by OGD and to determine the most effective dose. As shown in Figure 5A, 10^−6^ mM of liraglutide exerted the best protection. TUNEL staining was performed to explore whether liraglutide could inhibit neuronal apoptosis both *in vivo* and *in vitro*. *In vitro*, the control group showed low expression. The number of TUNEL-positive apoptotic cells was markedly decreased in the OGD + LIRA group compared with the OGD group. The OGD + Liraglutide + LY294002 group showed an increase in the number of TUNEL-positive apoptotic cells (Figures 7A,B). *in vivo*, the results showed the same trend as those obtained *in vitro*. However, the anti-apoptotic effect of liraglutide was inhibited by LY294002. The number of TUNEL-positive cells was significantly increased in the HI group 24 h after HI injury. In contrast, a significant reduction in the number of TUNEL-positive cells was detected in the HI + Liraglutide group. However, the number of TUNEL-positive cells was significantly increased in the HI + Liraglutide + LY294002 group compared with that observed in the HI + Liraglutide group, indicating that this protective effect was partially reversed by LY294002 (Figures 7C,D). Furthermore, the protein expression levels of Bcl-2 and cleaved caspase 3 were determined through western blotting. Quantitative data demonstrated that liraglutide reversed the HI-induced increase in the expression level of cleaved caspase 3 (Figure 7E). Meanwhile, the expression level of the anti-apoptotic protein Bcl-2 was significantly increased after treatment with liraglutide compared with that reported in the HI group (Figures 7E,F). In the HI + Liraglutide + LY294002 group, the level of Bcl-2 was decreased, whereas that of cleaved caspase 3 increased. These differences were statistically significant vs. those of the HI + Liraglutide group (Figures 7E–G). Collectively, these results indicate that liraglutide exerted an anti-apoptotic effect against HI injury, and the protective effect was partially reversed by LY294002 both *in vivo* and *in vitro*.

### Liraglutide Improved the Prognosis of Rats After Hypoxic–Ischemic Injury

**Figure 8:**
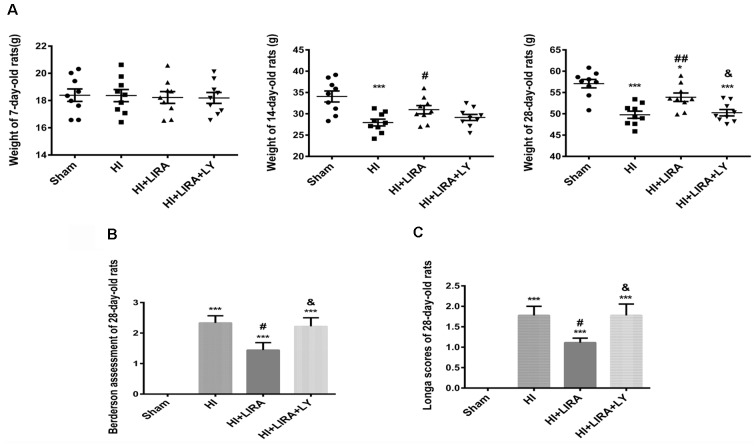
Liraglutide (LIRA) improved the prognosis of rats post HI injury. **(A)** Weights of rats on days 7, 14, and 28. *P < 0.05 and ***P < 0.001 vs. the sham group. ^#^*P* < 0.05 and ^##^*P* < 0.01 vs. the HI group. ^&^*P* < 0.05 vs. the HI + LIRA group. Values are presented as mean ± SEM. *n* = 9. Quantification data of the Berderson behavioral test **(B)** and Longa assessment **(C)** at day 28. ***P < 0.001 vs. the sham group. ^#^*P* < 0.05 and ^##^*P* < 0.01 vs. the HI group. ^&^*P* < 0.05 vs. the HI + LIRA group. Values are presented as mean ± SEM. *n* = 9.

The bodyweight of the rats was measured at 7, 14, and 28 days of age. Prior to HI injury (at day 7), the bodyweights did not show significant differences between the groups. However, the HI + Liraglutide group showed improvements in bodyweight at day 14 than in the HI group. However, there was no significant difference between the HI + Liraglutide group and HI + Liraglutide + LY294002 group at day 14. At day 28, the rats in the HI + Liraglutide group continued to exhibit improvements in bodyweight in contrast to the HI group, whereas the HI + Liraglutide + LY294002 group showed little improvements in contrast to the HI + Liraglutide group. Following long-term treatment with liraglutide, the HI + Liraglutide group exhibited greater weight gains than the HI group and the HI + Liraglutide + LY294002 group (Figure 8A).

Furthermore, at day 28, the Longa assessment and the Berderson behavioral test were performed to evaluate the injury and improvement of nerve function, respectively. At 28 days of age, these assessments indicated that the HI + Liraglutide group showed better mobility and flexion than did the HI group. In addition, the HI + Liraglutide + LY294002 group got higher scores, which revealed that the rats in this group did not perform well in these two tests (Figures 8B,C).

## Discussion

Neonatal HI brain injury, caused mainly by perinatal asphyxia, is associated with death and long-term neurological impairments in newborns. According to numerous animal studies and randomized clinical trials, therapeutic hypothermia is currently the standard treatment for this condition. However, approximately 50% the infants treated with hypothermia expire or suffer neurodevelopmental disability (Azzopardi et al., 2009; Liu and McCullough, 2013). Thus, it is important to develop new effective therapeutic approaches targeting the pathogenesis of HI brain injury through the processes of apoptosis, inflammation, oxidative stress, and excitotoxicity (Fernández-López et al., 2014).

Liraglutide has shown its unique neuroprotective effect in reducing the level of oxidative stress, apoptosis, and inflammation as well as in promoting the proliferation of neurons and the recovery of cognitive function in the central nervous system in experimental models and clinical patients (Zhu et al., 2016; Gejl et al., 2017; Guthrie, 2018). Moreover, liraglutide exerts positive effects on weight loss, blood pressure, hyperlipidemia, and glycemic control in the clinic. Thus, many studies have investigated its effects on the cardiovascular, endocrine, and nervous systems (Ishii et al., 2017; Babic et al., 2018; Bahtiyar et al., 2018). In animal models and clinical patients, there were no adverse events or reproductive toxicity found posttreatment with liraglutide. In this study, we did not observe obvious histopathological changes in other important organs after seven consecutive days of administration of liraglutide. Therefore, we suggest that liraglutide may be a safe therapeutic option for neonatal HI brain injury.

In our study, we found that liraglutide protected both short-term and long-term functions. Specifically, we found that treatment with liraglutide decreased the brain infarct volume and the degree of cerebral edema, inhibited inflammatory response and neuronal apoptosis, promoted the recovery of histomorphology, and improved the bodyweight and behavior disorders. Thus, the present data suggest a promising therapeutic strategy against neonatal HI brain injury.

Our results clearly showed that treatment with liraglutide improved neonatal brain injury in pups after HI. First, through TTC staining, we determined that the optimal concentration of liraglutide for a single intraperitoneal injection was 200 nmol/kg. Treatment with liraglutide reduced brain infarct volume 24 h after HI injury, showing evidence of a direct neuroprotective role. For the cerebral edema, significant enlargement of the ipsilateral brain in gross morphology could be observed as a result of HI injury. *in vitro*, we revealed that liraglutide promoted cell survival and suppressed cell apoptosis in the OGD model. After seven consecutive days of administration, liraglutide reduced brain tissue loss and attenuated brain atrophy. In addition, the results of the H&E, Nissl, and MAP-2 staining suggest that liraglutide maintained neuronal structure and function. MBP staining indicated the protective effect of liraglutide on the white matter. These results confirmed that liraglutide exerts short-term protective effects.

Previous studies have indicated that the consequences of neonatal HI brain injury are not temporary, leading to neuropathies associated with the activation of neuroinflammatory processes. To our knowledge, liraglutide exerts a favorable anti-inflammatory effect in many animal models (McClean and Hölscher, 2014; Wang et al., 2018). The level of inflammatory cytokines at both the mRNA and protein levels can be used to evaluate the inflammatory response. Our experiments detected the increased expression of these inflammatory factors in brain tissue following HI and clearly demonstrated that liraglutide plays an effective role in anti-neuroinflammation against neonatal brain injury by inhibiting the mRNA and protein expression in rats.

Current evidence suggests that activation of the PI3K/Akt and its downstream pathways suppresses neuronal apoptosis in models of HI brain injury (Tu et al., 2018) and under OGD conditions (Ye et al., 2019). GSK3β is a serine/threonine protein kinase with broad expression and one of the downstream targets of Akt. It is essential in energy metabolism (Martin et al., 2018) and nerve cell development (Chen et al., 2019a). The levels of GSK3β can be decreased by phosphorylation at the ser9 site (Xu et al., 2017; Chen et al., 2019b). Additionally, GSK3β (Ser9) can be phosphorylated by Akt, inhibiting its enzymatic activity. An increasing body of evidence indicates that activation of PI3K/Akt and inhibition of GSK3β play a neuroprotective role in many brain injury models, including experimental ischemic stroke (Valerio et al., 2011), subarachnoid hemorrhage-induced early brain injury (Ma et al., 2016), traumatic brain injury (Zhang et al., 2018), and intracerebral hemorrhage (Chen et al., 2019b). A recent study showed that liraglutide inhibits the apoptosis of osteoblastic MC3T3-E1 cells induced by serum deprivation partly through the PI3K/Akt/GSK3β signaling pathway (Wu et al., 2018). Hence, we investigated whether the mechanism of liraglutide involved in protecting against neonatal HI brain injury is related to the activation of the PI3K/Akt/GSK3β signaling pathway. Using the PI3K inhibitor of LY294002, we found that Akt phosphorylation was decreased in both the HI group and HI + Liraglutide + LY294002 group. In addition, treatment with liraglutide markedly increased the protein expression of activated Akt. Subsequently, liraglutide inhibited the activity of GSK3β by increasing the phosphorylation at the ser9 site. We observed that LY294002 could fully or partly reverse the protection offered by liraglutide in various manners, such as increasing the infarct volume and cerebral edema and reducing the prognostic effect, compared with those reported in the HI + Liraglutide group. Overall, we demonstrated that liraglutide exerted its neuroprotective effect by activating the PI3K/Akt/GSK3β signaling pathway.

Apoptosis plays an important role in HI brain injury (Zhu et al., 2005; Northington et al., 2011). Apoptosis is more pronounced in immature animal brains compared with juvenile and adult brains (Semple et al., 2013). Thus, the decrease of apoptosis has been considered a therapeutic target against HI injury in newborns. Numerous research studies reported that liraglutide suppresses apoptosis and promotes neurological functional recovery in some central nervous system diseases, such as traumatic brain injury (Li et al., 2015) and spinal cord injury (Chen et al., 2017). To the best of our knowledge, caspase 3 is one of the most representative indicators of apoptosis, and cleaved caspase 3 is released at high levels specifically following HI (Feng et al., 2014; Tu et al., 2019). The Bcl-2 family, especially Bcl-2, also protects neurons from apoptosis. It restricts the activity of caspase 3 and enhances cell survival. Our results, compared with those reported in the HI group, showed that liraglutide could downregulate the expression of cleaved caspase 3 and upregulate that of Bcl-2. Moreover, a decreasing number of TUNEL-positive cells was observed in rats with neonatal HI brain treated with liraglutide. Therefore, we confirmed that liraglutide plays a protective role through its anti-apoptotic activity after HI injury in newborn rats. However, LY294002 reversed the anti-apoptotic function of liraglutide both *in vitro* and *in vivo*. We illustrated that liraglutide inhibits neuronal apoptosis following HI and OGD by activating the PI3K/Akt/GSK3β signaling pathway.

This study was characterized by limitations. Several reports have demonstrated the neuroprotective effects of GLP-1R in terms of reducing oxidative stress and inhibiting apoptosis (Hamilton and Hölscher, 2009). Activation of GLP-1R stimulates adenylyl cyclase signaling, leading to an increase in the levels of cAMP that activate protein kinase A (PKA) and subsequently phosphorylate cAMP response element-binding protein (CREB). Activation of CREB regulates the transcription of the Bcl-2 proteins (Chen et al., 2017). In our study, the role of GLP-1R was not explored. Thus, as one of the downstream targets of Akt, mammalian target of rapamycin (mTOR) may also exert its influence on HI injury in newborns. Therefore, it is necessary to thoroughly investigate the possible underlying mechanism involved in this process. Additionally, a recent study showed that the protective effects of liraglutide may be associated with increased mTOR expression *via* activation of the AMPK and PI3K/Akt signaling pathways in rats with type 2 diabetes (Yang et al., 2018). Additional cell experiments are warranted to confirm the role of liraglutide. Considering the limitations of therapeutic hypothermia in the treatment of neonatal encephalopathy, it is vital to explore whether liraglutide augments hypothermic neuroprotection. Therefore, in our future study, a combination of liraglutide and hypothermia will be investigated.

## Conclusion

The present study demonstrated that treatment with liraglutide reduced infarct volume and ameliorated cerebral edema, decreased inflammatory response, promoted the recovery of tissue structure, and improved prognosis post HI insult *via* the PI3K/Akt/GSK3β signaling pathway. Furthermore, treatment with liraglutide inhibited apoptosis and promoted neuronal survival in both rat model and OGD insult experiments. The pathogenesis of neonatal HI brain injury is complicated, and effective treatment methods are currently limited. Therefore, liraglutide may be a promising and inexpensive therapeutic option in this setting. Further studies are warranted to confirm the mechanism.

## Data Availability Statement

The datasets of our study are available from the corresponding author upon reasonable request.

## Ethics Statement

The animal study was reviewed and approved by the Animal Care and Use Committee of Wenzhou Medical University (ethic number: wydw2014-0058).

## Author Contributions

SZ and JB drafted the article and designed the study mainly. HJ helped to design experiments. JZ and CF wrote part of the article. MH and JZ designed figures and contributed to analysis of part of the data. PL, XF and SC helped to analyze related data. ZL contributed to critical revision of the manuscript. All the authors approved the final version and agreed to be accountable for the study.

## Conflict of Interest

The authors declare that the research was conducted in the absence of any commercial or financial relationships that could be construed as a potential conflict of interest.
