# Supplementary material for: Treatment With Liraglutide Exerts Neuroprotection After Hypoxic–Ischemic Brain Injury in Neonatal Rats via the PI3K/AKT/GSK3β Pathway
Source: Front Cell Neurosci. 2020 Jan 30;13:585. doi: 10.3389/fncel.2019.00585 (PMC7003644; doi:10.3389/fncel.2019.00585)
Supplement: Supplementary file 1 [file Data_Sheet_1.PDF]

Supplementary Material

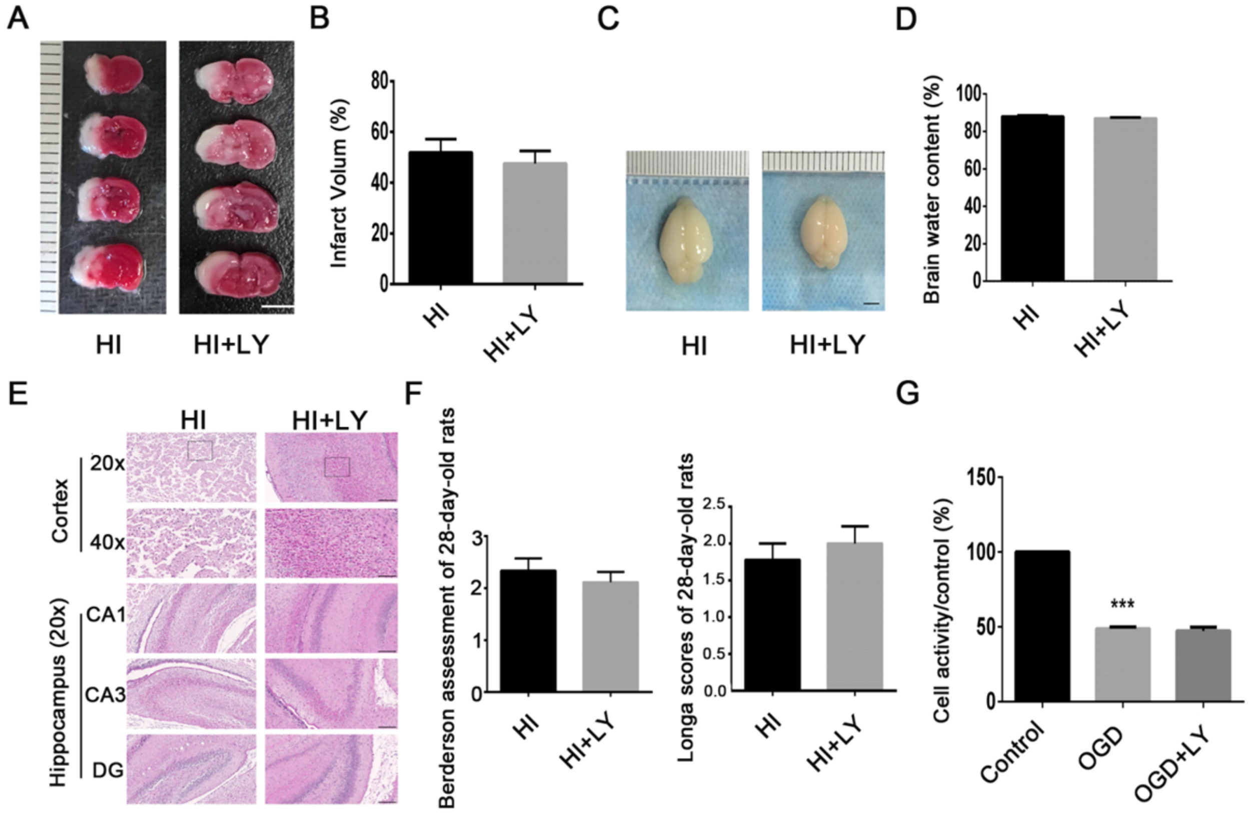

Supplementary Figure 1. The comparion between the HI group and the HI+LY294002 group. (A) Representative TTC-stained coronal brain sections 24 h after HI. n = 4. Scale bar = 1 mm. (B) Quantitative analysis of infarct volume. Values are presented as mean ± SEM. n = 4. (C) The general shape of the brain 24 h after HI. n = 5. Scale bar = 1 mm. (D) Quantification of water content in the ipsilateral brain hemisphere 24 h after HI. Values are presented as mean ± SEM. n = 5. (E) Representative images of H&E staining in the cortex and hippocampus of CA1, CA3, and dentate gyrus at day 7 post HI injury. Scale bar = 25 µm, 50 µm. (F) Quantification data of the Longa assessment and Berderson behavioral test at day 28. Values are presented as mean ± SEM. n = 9. (G) Cell viability after reoxygenation for 24 h following OGD. ***P<0.001 versus the control group. Values are presented as mean ± SEM. n = 3.
